# The impact of educational digitalization on life satisfaction: an empirical analysis based on PISA 2022

**DOI:** 10.3389/fpsyg.2026.1740535

**Published:** 2026-01-29

**Authors:** Xu Fang, Zejin Mo

**Affiliations:** College of Educational Sciences, Nantong University, Nantong, China

**Keywords:** anxiety, educational digitalization, learning interest, life satisfaction, PISA 2022

## Abstract

**Introduction:**

The continuous and in-depth development of educational digitalization has become a key education development strategy for many countries around the world. Current research has paid considerable attention to the relationship between educational digitalization and educational outcomes, but the impact of educational digitalization on life satisfaction remains underexplored, lacking in-depth theoretical analysis and empirical evidence.

**Methods:**

The article first constructs a theoretical model of the impact of educational digitalization on students’ life satisfaction based on relevant theories, followed by an empirical analysis. Large-scale cross-national data from PISA 2022 is used to empirically examine the direct and indirect effects of educational digitalization on students’ life satisfaction.

**Results:**

The results indicate a significant positive effect of educational digitalization on students’ life satisfaction. Specifically, learning interest and learning anxiety play significant mediating and chain-mediating roles in this relationship. This suggests that digitalization can enhance students’ well-being by stimulating their interest in learning and alleviating learning anxiety. Furthermore, grade level significantly moderates this effect: among junior high school students, the pathway through interest and anxiety in improving life satisfaction is more pronounced.

**Discussion:**

Educational digitalization has a direct impact on students’ life satisfaction, while interest and anxiety play mediating and chain-mediating roles in the effect of educational digitalization on life satisfaction. At the same time, grade level plays a moderating role in the impact of educational digitalization on students’ life satisfaction. This study deepens the understanding of the mechanisms by which digitalization promotes student well-being and emphasizes that strategies for digital education should focus on stimulating interest and managing emotions, while implementing differentiated interventions for students of different age groups.

## 1 Introduction

In the era of rapid information technology development, the wave of digitalization has penetrated all aspects of social life with unprecedented depth and breadth, eliciting widespread attention and reflection. In the field of education, educational digitalization represents a new paradigm that aligns with the trends of the times. From flipped classrooms to virtual laboratories, and from learning management systems to AI-assisted teaching tools, digital technologies have permeated every dimension of education, reshaping traditional modes of learning and instruction. This transformation has not only altered the way teaching content is delivered but has also profoundly influenced teacher–student interaction, students’ learning pace and learning experience. Globally, developed countries such as those in Europe and North America took an early lead in developing educational information systems and digital infrastructure, thereby establishing relatively mature digital education ecosystems (Voronkova et al., 2023). In China, with the progressive implementation of the Strategic Action Plan for Educational Digitalization, the focus has shifted from mere resource construction to deep pedagogical integration, promoting substantial improvements in educational equity and quality (Wu, 2023). Particularly during the COVID-19 pandemic, digital learning environments rapidly evolved from supplementary instructional tools into primary learning modalities, accelerating the digital transformation of education. The scope of educational digitalization is extensive, encompassing not only the widespread use of hardware such as computers, Internet connectivity, and interactive whiteboards in schools and homes, but also the development and utilization of software resources, including digital textbooks, online learning platforms, educational applications, and diverse digital resource repositories. Educational digitalization is driving the shift from traditional teacher-centered instruction to student-centered personalized learning, inquiry-based learning, and collaborative learning. The Organization for Economic Cooperation and Development (OECD) has consistently highlighted the role of digital technologies and skills in educational innovation in its reports (Dong and Shao, 2017), and has encouraged its member countries to leverage technology to improve the quality of learning outcomes and educational equity. This transformation goes beyond the mere application of technological tools, encompassing a systemic reform of educational philosophy, curriculum design, teaching methodologies, and assessment systems.

As the digitalization of education sweeps across the globe, students’ life satisfaction—an essential indicator of their subjective and overall well-being—has increasingly drawn widespread attention from educational researchers, policymakers, and the public alike (Pan and Cutumisu, 2023; Zhang et al., 2024). Life satisfaction reflects an individual’s cognitive appraisal and emotional experience regarding their quality of life and overall well-being. For adolescent students, who are in a critical stage of physical and psychological development, high life satisfaction is not only closely associated with a positive mental state and healthy physical growth but also exerts a profound positive influence on their academic achievement, social adaptation, and future success and happiness (Orben et al., 2019). Student life satisfaction is shaped by a complex interplay of factors, including individual-level factors (such as personality traits, self-efficacy, and coping styles), family-level factors (such as parental support and family socioeconomic status), school-level factors (such as teacher–student relationships, peer relationships, school climate, teaching quality, and academic pressure), and broader sociocultural environmental factors (Michaud, 2006; Aldridge et al., 2019; Gedutienė and Lukšaitė-Samaitienė, 2018). In recent years, with the increasing integration of digital technology into students’ learning and daily lives, the potential impact of digital education on student’ life satisfaction—an emerging yet crucial environmental factor—has become an issue that warrants urgent and in-depth scholarly investigation.

The impact of educational digitalization on students’ life satisfaction may be both multidimensional and multidirectional, shaped by numerous moderating factors. On the one hand, digital technology provides unprecedented opportunities for education. It offers students richer and more accessible learning resources, removes temporal and spatial constraints, and facilitates personalized and autonomous learning, thereby enhancing learning motivation and a sense of achievement, ultimately improving life satisfaction. Digital tools and platforms can also promote collaboration and communication among students, broaden their social networks, and strengthen their sense of belonging. Furthermore, acquiring digital skills is regarded as essential for improving individual competitiveness and adaptability, enabling students to better position themselves and flourish in the digital era, which may also positively influence their life satisfaction. For example, several studies have demonstrated that the effective use of digital technology can enhance students’ autonomy, sense of competence, and sense of belonging within the learning community (Zhu et al., 2024; Folta et al., 2022; Jones et al., 2018), all of which are crucial factors contributing to overall well-being.

On the other hand, the deepening advancement of digital education may also be accompanied by potential risks and challenges that negatively affect students’ life satisfaction. Extended periods of screen time for learning and entertainment may lead to a lack of physical exercise among students, thereby increasing the risk of health issues such as obesity. At the same time, it may also contribute to physiological problems like vision deterioration and insufficient sleep. All of these factors may indirectly reduce life satisfaction. Excessive reliance on digital technology for communication may reduce opportunities for face-to-face social interaction, hinder the development of social skills and the formation of meaningful interpersonal relationships, and even lead to the emergence of a sense of social isolation. Cybersecurity concerns—such as cyberbullying, exposure to harmful online content, and breaches of personal privacy—can also cause psychological stress and distress among students, undermining their emotional health and overall well-being. Moreover, the digital divide, referring to disparities in access to digital devices, internet connectivity, and digital literacy among students from different regions, schools, and socioeconomic backgrounds, may also result in unequal educational opportunities and exacerbate social inequality, thereby diminishing the life satisfaction of disadvantaged students (Kim et al., 2021; Li et al., 2022). Meanwhile, the existence of the digital divide may also lead to inadequate development of emotional intelligence, increased anxiety levels, and more behavioral problems among students in the context of digitalized education.

Therefore, the relationship between educational digitalization and students’ life satisfaction cannot be reduced to a simple linear correlation. It is, rather, a complex issue that requires in-depth analysis through rigorous empirical research. Understanding the nature, strength, mechanisms, and influencing factors of this relationship is crucial. Such understanding provides vital theoretical insights and practical significance for formulating sound digital education policies, optimizing the application of digital technologies in teaching, and ultimately enhancing student well-being by effectively harnessing the positive effects of educational digitalization while mitigating its potential risks. The Programme for International Student Assessment (PISA), a globally influential international student literacy assessment program initiated by the Organization for Economic Cooperation and Development, provides a valuable research opportunity. PISA not only assesses student literacy in core subject areas such as reading, mathematics, and science, but also collects extensive data on student backgrounds, learning experiences, school environments, and ICT use through the way of student and school questionnaires (Xu et al., 2025). It is particularly noteworthy that the PISA 2022 questionnaire includes a dedicated module on student well-being, which incorporates specific indicators for measuring students’ life satisfaction. This feature makes it possible to systematically analyze the impact of educational digitalization on students’ life satisfaction using the large-scale, cross-national dataset provided by PISA 2022. This study aims to construct an appropriate analytical model based on the latest PISA 2022 data to empirically examine the relationship between digital education and students’ life satisfaction. Through this research, we hope to provide insightful insights and evidence-based policy recommendations on how to promote student well-being in the context of global digital education. Building on the above analysis, the following sections will proceed in two steps. First, we will synthesize existing research on the impact of educational digitalization on students’ life satisfaction and delineate the limitations or areas warranting further exploration in prior studies. Subsequently, based on this foundation, we will conduct an in-depth theoretical and empirical investigation.

## 2 Literature review

Existing research indicates that the impact of digital education on students’ academic performance and life satisfaction has become a key topic in international educational research. Overall, international studies began relatively early, developing a more systematic theoretical framework and a multidimensional empirical research system. Domestic research in this field started relatively late. However, driven by the ongoing promotion of national educational informatization policies, it has developed rapidly and is gradually evolving into a research landscape that is distinctly practice-oriented and characterized by indigenous features.

International research on the relationship between digital technology and student life satisfaction continues to gain traction, gradually expanding beyond general digital technology use to encompass digital learning experiences in educational contexts. Numerous experts have found that the appropriate use of online learning tools can enhance students’ learning autonomy, sense of competence, and sense of belonging. These enhanced autonomy, competence, and sense of belonging, in turn, extend to everyday life, becoming crucial support for students to build positive self-perceptions and cope with life’s challenges, ultimately contributing to increased life satisfaction (Masrek et al., 2024; James et al., 2021). However, excessive or low-quality use of digital technologies may lead to the disorderly influx of massive information, which can easily trap students in the dilemma of “information overload.” This makes it difficult for them to filter core knowledge and reduces learning efficiency. If such low-quality usage persists over the long term, it may also induce dual psychological burdens. On the one hand, anxiety may arise due to learning outcomes falling short of expectations; on the other hand, prolonged mechanical engagement with technology may lead to cognitive exhaustion, further triggering learning burnout. All of these factors thereby negatively impact students’ mental health and the sustainability of their learning development (Wang F. et al., 2024; Wang S. et al., 2024; Shkembi et al., 2024). These findings suggest that factors related to educational digitalization influence student well-being through complex psychological and social mechanisms, with multidimensional and interactive effects. In contrast, domestic research on the relationship between educational digitalization and student life satisfaction is still in its infancy, with much of the focus on the psychological and social issues associated with digital technology use. Existing studies primarily focus on university students, exploring the impact of internet addiction, social media dependence, and anophthalmia on life satisfaction, social connectedness, and academic resilience. Research has found that the level of social media addiction can significantly predict the occurrence of nomophobia, while digital addiction negatively impacts social connectedness and academic resilience. In turn, social connectedness exerts a significant positive effect on life satisfaction (Cheng and Wu, 2024; Zhang et al., 2021). Overall, domestic research has predominantly focused on the potential negative impacts of digital usage, while paying insufficient attention to the positive effects of educational digitalization on enhancing students’ learning experiences, personalized development, and psychological well-being. Furthermore, there is a lack of systematic theoretical construction and empirical examination regarding its underlying mechanisms and mediating pathways.

In the research on educational digitalization based on PISA, most of the focus is on the research on educational digitalization and academic performance, international scholars have been early on to systematically explored the impact of digital education on student academic achievement using large databases such as the Program for International Student Assessment. Overall, the impact of digital technology use on academic performance exhibits significant complexity and nonlinearity. Moderate, learning-oriented digital learning behaviors can contribute to the improvement of students’ academic outcomes. Maria Cornachione Kula et al., in an empirical analysis based on PISA 2015 data, found that learning-oriented digital device use outside of school is positively correlated with science scores, while excessive use within school may negatively impact academic performance (Dong and Kula, 2022; Huang et al., 2019). Hu and Yu (2021) based on PISA 2018 data, further found that students’ positive attitudes toward digital learning were significantly positively correlated with digital reading scores, while ICT-based social media use was negatively correlated with reading scores. The latest PISA 2022 study results indicate that both the time students spend using digital resources for learning mathematics and the extent of their out-of-school digital learning activities are significant predictors of their mathematical performance (Joshi et al., 2025). These studies collectively reveal that the impact of educational digitalization on students’ academic achievement exhibits a distinct context-dependent nature, meaning its effects are influenced by multiple factors such as usage scenarios, purposes, frequency, and subject-specific differences. In contrast, domestic research based on PISA data has primarily focused on analyzing the current status of students’ information literacy and ICT competence and exploring the influencing factors. Existing studies have shown that the ICT competence of students in four provinces and municipalities in China (Beijing, Shanghai, Jiangsu, and Zhejiang) is relatively low overall among participating countries and regions, and that variables such as household ICT resources, ICT usage in schools and at home, and student interest all have a significant positive impact on ICT competence (Jeong et al., 2024; Wang F. et al., 2024; Wang S. et al., 2024; Huang et al., 2021; Huang et al., 2019). This type of research emphasizes the need to further improve the ICT education system, strengthen information literacy assessment mechanisms, and enhance students’ interest in information technology learning. Overall, domestic research has made some progress in the development of digital education capabilities and educational practices, but there is still a lack of in-depth and systematic empirical analysis of the interaction between teaching behavior, learning processes, and academic performance in digital learning environments.

A comprehensive review of the relevant research literature at home and abroad reveals that the impact of digital education on student development has become a global research hotspot, with fruitful results. However, significant research gaps remain in the following areas, providing an opportunity for this study:

First, there is a lack of empirical research directly focusing on the relationship between digital education and student life satisfaction. While numerous studies have explored the impact of digital technology on students’ academic achievement, specific skills (such as digital reading literacy), or mental health (such as internet addiction and anxiety), and some research has begun to focus on students’ overall well-being or life satisfaction and the factors influencing it, there is a lack of empirical research that uses “digital education” as a core independent variable and systematically examines its direct impact on the core dependent variable of “student life satisfaction “.

Second, the mechanisms and boundary conditions for the impact of digital education on students’ life satisfaction remain insufficiently explored. The impact of digital education on students’ life satisfaction may not be direct, but rather achieved through a series of mediating variables (such as learning motivation, academic engagement, teacher-student interaction, peer relationships, digital skills, and self-efficacy). It may also be influenced by multiple moderating variables (such as individual student characteristics, family background, school culture, and the level of digital development in the country or region). While existing studies have sporadically mentioned several potential mediating or moderating factors, there remains a lack of systematic theoretical frameworks and empirical testing to clarify the mechanisms through which such complex pathways and conditional effects operate.

It is evident that current research on the impact of educational digitalization on life satisfaction still suffers from insufficient empirical evidence and a lack of in-depth analysis regarding its underlying mechanisms. Therefore, the following section will first construct a theoretical model of how educational digitalization affects students’ life satisfaction—incorporating mediating and moderating effects—based on relevant theories, and then proceeds to conduct a thorough empirical analysis accordingly.

## 3 Construction of the model

UNESCO notes that digital literacy has become a core competency for 21st-century citizens, influencing individuals’ participation and adaptability in the digital society. Educational digitalization is not a one-dimensional application of technology, but rather a systemic transformation process comprising three key dimensions: digital infrastructure, quality of digital use, and student digital competence. Firstly, digital infrastructure serves as the material foundation of educational digitalization, referring to the totality of hardware, network environments, platform systems, and digital resources that support digital teaching and learning, such as campus networks, interactive terminals, learning management systems, and digital content repositories. Secondly, the quality of digital use represents the procedural core of educational digitalization. It transcends the mere presence of technology and focuses on the effectiveness of deep integration between digital tools and educational practices. This includes innovative applications of digital tools and resources in teaching, the design of interactive learning experiences, and data-driven precision instruction and assessment. Finally, student’ digital competence embodies the key literacy and intended outcome of educational digitalization. It denotes the comprehensive ability of students to responsibly, critically, and creatively use digital technologies for information processing, communication and collaboration, problem-solving, and content creation to meet the demands of learning, daily life, and personal development. Life satisfaction is an individual’s positive psychological experience and subjective judgment derived from cognitively assessing their overall life conditions or specific life domains (such as health, interpersonal relationships, career, finances, etc.) based on self-defined criteria. It reflects the individual’s perception of the alignment between their current life situation and their expectations, serving as one of the core indicators for measuring subjective well-being. According to the Technology Acceptance Model (TAM), an individual’s proficiency in technology significantly impacts their user experience and effectiveness. This theory emphasizes that when users perceive a technology as easy to use and useful, they develop a more positive attitude and a higher willingness to use it. For example, research shows that students with strong digital literacy are more inclined to use AI-assisted tools (such as Grammarly and ChatGPT) to optimize their writing, describing them as “user-friendly and intuitively functional” (Hadi Mogavi et al., 2024), thereby enhancing learning efficiency. This perception of ease of use encourages students to use technology more frequently, fostering a positive attitude toward its adoption. Additionally, students with higher digital proficiency can effectively leverage digital tools to expand their learning resources. For instance, they can utilize academic search engines (such as Google Scholar and Web of Science) to precisely locate cutting-edge literature or turn to specialized forums (such as Stack Overflow or academic subforums on Reddit) to solve programming challenges. A survey of university students found that students skilled in using digital resources achieved significantly better grades in course projects, validating the perceived usefulness of technology (Mei et al., 2019). Students with a high level of digital literacy are better able to utilize digital tools to access learning resources, expand social networks, and participate in recreational activities, thereby improving their overall quality of life.

From the perspective of resource acquisition, students with strong digital skills are able to search, filter, and utilize online information resources more efficiently, thereby enabling them to quickly find solutions when encountering difficulties in the learning process (Graciano and Benablo, 2023; Yu et al., 2024). Information processing theory emphasizes that effective information retrieval and processing are key factors for success in modern society. This enhanced problem-solving ability enhances students’ self-efficacy (Ningsih and Hayati, 2020), giving them greater confidence and motivation to face challenges. Therefore, this study proposes that students with higher digital skills are more likely to fully utilize the convenience and opportunities offered by digital technology, gain positive experiences across multiple dimensions and thus achieve higher life satisfaction. This hypothesis not only has a theoretical basis but also aligns with the actual experiences and developmental needs of the digital native generation. Students with a high level of digital proficiency can more fully utilize existing digital resources. Through the rational use of these resources, they achieve better learning outcomes, which in turn enhance their life satisfaction. For instance, students with stronger digital skills can effectively leverage generative artificial intelligence to clarify doubts (Ouyang et al., 2025), access personalized learning resources (Wu and Wu, 2024), and engage in inquiry-based learning (Zheng et al., 2024), thereby improving both their learning effectiveness and personal life experiences. Additionally, digitally proficient students can more thoroughly utilize online educational resources, such as MOOCs (Liu and Han, 2023), to enhance their learning quality, broaden their perspectives, and expand their learning networks, thus further increasing their life satisfaction. Based on this research, we propose Hypothesis H1.

*H1*: Digitalization level has a positive impact on students' life satisfaction.

According to Self-Determination Theory, intrinsic motivation is a crucial factor influencing individual well-being. As a key component of intrinsic motivation, interest plays a vital role in an individual’s psychological health and life satisfaction (Lydon-Staley et al., 2019). Digital technology, with its interactive, personalized, and multimedia features, can effectively stimulate students’ interest in learning and desire for exploration. First, digital learning environments provide a rich variety of learning resources and presentation format that cater to the needs of students with different learning styles. Digital learning environments enhance students’ sense of autonomy by providing personalized customization and adaptive learning pathways, thereby stimulating their intrinsic learning motivation. For example, through data analysis and artificial intelligence technologies, teachers can identify students’ learning patterns, predict potential difficulties, and offer targeted additional support (Surur et al., 2024). This includes tailored course plans and real-time adjustments to learning resources, enabling students to learn at their own pace and delve deeper into topics of interest. Research indicates that personalized learning activities effectively fulfill students’ need for autonomy, consequently boosting their intrinsic motivation (Alamri et al., 2020). For instance, in art and design education, teachers who support students’ autonomy while integrating digital learning tools can significantly enhance students’ learning engagement (Tao, 2025). Adaptive learning systems analyze student behavior through real-time data and promptly adjust learning resources and pathways to meet students’ autonomy needs. Multiple intelligence theory suggests that different students possess distinct intellectual strengths, and digital technology provides a platform for learners of all types to showcase and develop their talents (Cavas and Cavas, 2020; Statti and Torres, 2020). For example, artificial intelligence technology can provide students with personalized guidance and assign customized exercises, meeting their needs for individualized learning and thereby enhancing their interest in learning (Sun and Zhang, 2025). Similarly, students can learn through MOOC courses anytime and anywhere at their own pace (Zhang, 2024), while the fragmented format of micro-lectures also makes learning highly flexible (An et al., 2017), further significantly boosting students’ learning motivation. Second, the immediate feedback provided by digital learning tools can enhance students’ sense of engagement and accomplishment, stimulating their continued interest in learning (Wong and Yang, 2017; Fanshawe et al., 2020; Ajogbeje, 2023). Methods such as real-time scoring in online quizzes, progress bars, and achievement systems in gamified learning enable students to immediately perceive their learning progress. This reinforces the belief of “I can do it,” reduces frustration, and sustains learning interest. For example, the application of polling technology in physics teaching has effectively enhanced students’ metacognitive abilities through instant feedback (Molin et al., 2020). Immediate feedback, whether cognitive, emotional, or metacognitive, can enhance students’ learning motivation and self-efficacy by fulfilling their need for a sense of competence. Lastly, collaborative platforms and digital tools promote student interaction, fulfill their social needs, and make the learning process more enjoyable and meaningful. For instance, online discussion forums and virtual groups encourage collaborative learning and knowledge sharing among students. These approaches effectively enhance students’ behavioral and emotional engagement in learning, as they provide richer interaction and immersive experiences (Jeong et al., 2022), thereby fostering learning motivation and engagement. When students’ interests are stimulated and fulfilled, positive emotional experiences are generated. These positive emotions can then spill over into other aspects of life, thereby enhancing overall life satisfaction. The higher the level of students’ digital proficiency, the more the learning environment can support autonomous choice, competence development, and social connection, thereby significantly enhancing students’ intrinsic motivation. This is reflected in greater learning interest, which acts as a mediating variable, transforming the positive experiences of digital learning into sustained positive emotions and a sense of self-fulfillment. These psychological resources ultimately contribute to improving students’ overall life satisfaction. Therefore, this study proposes that interest plays a mediating role between digitalization level and life satisfaction. Based on the above research, we propose hypothesis H2.

*H2*: Digitalization level has a positive impact on life satisfaction by affecting students' interest.

Learning anxiety refers to a state of emotional distress experienced by individuals during the learning process due to excessive worry and pressure regarding learning tasks, outcomes, or their own abilities. It is typically manifested as tension, fear, self-doubt, avoidance of learning-related behaviors, and is often accompanied by physiological reactions such as insomnia and difficulty concentrating. According to stress and coping theory, an individual’s coping resources when facing environmental challenges influence their perceived stress and mental health. In the digital age, students face challenges such as rapid technological advancement and information overload. Lack of relevant digital skills can lead to increased technological anxiety and academic stress (Wong and Yang, 2017; Fanshawe et al., 2020; Ajogbeje, 2023). Students with higher digital proficiency possess stronger technological adaptability and problem-solving abilities, enabling them to cope with digital learning tasks with ease and reduce the frustration and anxiety caused by technological barriers. Furthermore, proficient digital skills improve learning efficiency, reduce the time and effort required to complete tasks, and thus reduce academic stress. For example, when students encounter difficulties in their studies, they can seek assistance from generative artificial intelligence to obtain explanations of knowledge or improve their learning approaches (Qi and Zhou, 2024). This helps them develop greater confidence in their learning, thereby reducing academic stress or anxiety. Additionally, digital skills can help students strengthen their sense of control over the learning environment, thereby reducing anxiety caused by environmental uncertainties. According to the Broaden-and-Build Theory of Emotions, negative emotions tend to narrow an individual’s scope of thinking and behavior, while positive emotions can broaden cognitive and behavioral resources (Fredrickson, 2001, 2004, 2013). Anxiety, as a typical negative emotion, significantly affects an individual’s life satisfaction. Anxiety possesses a narrowing effect that compels individuals into defensive cognitive patterns, limiting exploratory behaviors and social connections. This in turn hinders the accumulation of psychological capital and reduces life satisfaction (Yetim et al., 2024). In contrast, positive emotions can broaden an individual’s cognitive flexibility and behavioral possibilities. When students effectively handle learning tasks by leveraging their digital skills, technology use is no longer a threat but becomes an empowering tool. This allows students to more easily gain a sense of achievement and experience positive emotions. Such a positive emotional state can trigger a virtuous “broaden-and-build” cycle, enhancing an individual’s cognitive flexibility and promoting the continuous accumulation of social and psychological resources, ultimately leading to a significant improvement in life satisfaction (Li et al., 2025). This demonstrates that digital proficiency not only directly constitutes the foundation of an individual’s ability to adapt to the digital environment but also indirectly promotes life satisfaction through the psychological pathway of alleviating anxiety. This pathway aligns with the logical chain of “resources → psychological state → adaptation outcomes.” Specifically, digital proficiency reduces anxiety levels, thereby releasing the inhibitory effects of negative emotions on cognition and behavior, and subsequently activating positive psychological mechanisms that enhance life satisfaction. Therefore, digitalization can improve students’ life satisfaction by reducing their anxiety levels. Based on the above research, we propose hypothesis H3.

*H3*: Digitalization level has a positive impact on life satisfaction by affecting students' anxiety.

According to emotion regulation theory, positive and negative emotions influence each other, with positive emotions effectively regulating and alleviating the effects of negative emotions (Carr et al., 2021). As a positive emotional state, interest can not only directly promote an individual’s psychological health but also indirectly influence their well-being by reducing negative emotions. When students develop a strong interest in learning content or activities, they enter a state of positive affect, which is characterized by the following aspects. First, a state of high interest can enhance students’ concentration and cognitive engagement, making them more likely to experience a state of “flow” (Shirey and Reynolds, 1988; Fernes and Dengel, 2023). For example, metaverse technology enables students to immerse themselves, actively engage, and experience the joy of acquiring knowledge (Fan and Jin, 2025). Second, interest-driven learning activities lead to more successful experiences and positive feedback, enhancing students’ self-confidence. Secondly, interest-driven learning activities lead to more successful experiences and positive feedback, thereby enhancing students’ self-confidence. For example, students who engage in AI-assisted personalized learning significantly increase their interest in learning and develop greater confidence in their learning outcomes, which results in a notable decrease in their anxiety levels (Liu B. Q. et al., 2024; Liu Q. T. et al., 2024). Finally, positive emotional states promote the brain’s secretion of neurotransmitters such as dopamine, physiologically reducing anxiety and stress perceptions. When anxiety levels decrease, students are able to view life more positively and better cope with challenges, thereby enhancing their satisfaction with various aspects of life. This positive psychological state helps individuals develop a more optimistic attribution style and strengthens their hope and expectations for the future, ultimately contributing to an increase in life satisfaction. Therefore, this study proposes a chain mediation model: digitalization level first stimulates students’ learning interest, enhancing their positive emotional experiences; increased interest further reduces students’ anxiety and stress perceptions; and ultimately, reduced anxiety levels contribute to increased students’ life satisfaction. This chain mediation effect reveals the deep psychological mechanism by which digitalization level affects life satisfaction. Based on the above research, we propose hypothesis H4.

*H4*: Digitalization level affects students' anxiety by affecting their interest, thus having a positive impact on life satisfaction.

From the perspective of usage preferences, there are significant gender differences in the use of digital technology between male and female students. Male students tend to use digital technology more for gaming entertainment, technological exploration, and competitive activities, which can satisfy their needs for a sense of achievement and control (Lucas and Sherry, 2004). Female students, on the other hand, are more likely to use digital technology for social communication, information sharing, and collaborative learning, activities that are more aligned with their relationship-oriented values (Boneva et al., 2001). This difference in usage preferences leads to significant differences in the path and intensity of the impact of digital technology on life satisfaction among students of different genders. From the perspective of psychological need satisfaction, self-determination theory posits that individuals’ basic psychological needs include autonomy, competence, and relatedness (Ryan and Deci, 2000). Male students tend to seek satisfaction in autonomy and competence through digital technology, such as achieving a sense of accomplishment through activities like programming, game strategies, and technological innovation. Female students, on the other hand, tend to seek satisfaction in relational needs through digital platforms, such as maintaining friendships, participating in online communities, and expressing emotions through social media (Sheldon et al., 2001). This difference in the way these psychological needs are satisfied moderates the effect of digital literacy on life satisfaction. Research indicates that there are certain differences between male and female students in their use of educational technology, with one key factor being that males generally exhibit higher self-efficacy than females (Xue, 2015; Nelson-Le Gall and DeCooke, 1987). A study on gender differences in computer self-efficacy among university students revealed that males outperformed females in terms of computer experience, attitudes, and self-efficacy (Marakas et al., 2011). This disparity may stem from societal expectations that encourage males to demonstrate a sense of mastery over technology. Higher self-efficacy may enable males to view digitally advanced environments as opportunities for exploration, thereby more effectively stimulating their intrinsic motivation and significantly enhancing their life satisfaction. Scholars have observed gender differences in technology application within MOOC education, potentially influenced by variations in social roles between males and females (Fang and Yang, 2016; Morris and Venkatesh, 2000). Males may exhibit greater acceptance of technology and more positive engagement in its application (Yu et al., 2024). For instance, in the learning and application of artificial intelligence technology, males tend to report higher satisfaction (Borwein et al., 2023; Skalka et al., 2025). Gender differences may also moderate the intensity of anxiety induced by digitalization. Studies suggest that females typically display higher levels of computer anxiety or sensitivity to information overload, referred to as technostress (Russo et al., 2025), which describes the negative impact of technology on attitudes, thoughts, behaviors, or physiology. Research by Akkaş and Turan (2025) based on the Stimulus–Organism–Response (S-O-R) model, indicates that female users of social media platforms like Instagram are more susceptible to anxiety and burnout due to continuous information flow, leading to cognitive overload and reduced attention and decision-making efficiency. This suggests that as digital proficiency increases, females may be more prone to experiencing stress from technological complexity or concerns about missing information. Such digitally induced anxiety can increase cognitive load, thereby partially counteracting the positive effects of digitalization and potentially inhibiting life satisfaction. Therefore, this study proposes that gender significantly moderates the relationship between digital proficiency and life satisfaction, with students of different genders potentially exhibiting varying strengths of this effect. Based on this, the following hypothesis is proposed:

*H5*: Gender plays a moderating role in the positive impact of digitalization level on students' life satisfaction.

*H6*: Gender plays a moderating role in the positive impact of digitalization level on life satisfaction by affecting students' interest.

*H7*: Gender plays a moderating role in the positive impact of digitalization level on life satisfaction by influencing students' anxiety.

*H8*: Gender plays a moderating role in the positive impact of digitalization level on life satisfaction by affecting students' interest and then affecting their anxiety.

Relevant studies indicate that younger students are in a critical transition period from the concrete operational stage to the formal operational stage, during which their abstract thinking abilities and metacognitive skills are still developing. For students in this age group, digital technology primarily serves as concrete tools and gaming platforms, with its impact mainly reflected in direct user experiences and immediate gratification (Subrahmanyam and Greenfield, 2008). Research by Tobin and Grondin indicates that 14- to 15-year-old adolescents tend to significantly underestimate the passage of time while playing video games such as Tetris. This suggests that they are more prone to entering a flow state during immersive digital activities but lack the ability to actively monitor and strategically regulate their learning goals (Tobin and Grondin, 2009). Older students, with more mature abstract thinking skills, are better able to utilize digital technology for complex learning activities, creative expression, and social participation. Therefore, the impact of digital technology on their life satisfaction may be more profound and lasting. From the perspective of social development, students of different ages face different developmental tasks. According to Erikson’s theory of psychosocial development, middle school students primarily face conflicts over identity and role confusion, while high school students are more focused on building close relationships and planning for the future (Mitchell et al., 2021; Kadhim, 2025; Tettey et al., 2023). This difference in developmental tasks leads to significant differences in the needs and use of digital technology among students of different ages. Younger students may primarily use digital platforms to explore their self-identity and pursue personal interests, whereas older students tend to utilize digital tools more frequently for academic planning, career exploration, and deeper social interactions (Pérez-Torres, 2024; Murad, 2024). Research indicates that increasing age may lead to a decline in the ability of older adults to process complex stimuli and allocate attention in work settings compared to younger individuals (Plude and Hoyer, 1985). These capabilities are often necessary for the educational application of digital technologies. From the perspective of technology acceptance, although contemporary students are generally referred to as “digital natives,” differences in technology acceptance and depth of use exist among students of different ages. Older students typically possess stronger learning strategies and self-regulation abilities, enabling them to more effectively utilize digital technologies for in-depth learning and innovative practices (Prensky, 2001). This difference in ability moderates the effect of digital literacy on life satisfaction. Therefore, this study posits that age significantly moderates the impact of digital proficiency on life satisfaction, with students at different developmental stages potentially exhibiting varying sensitivity and response patterns. Based on the above analysis, we propose the following hypotheses:

*H9*: Age plays a moderating role in the positive impact of digitalization level on students' life satisfaction.

*H10*: Age plays a moderating role in the positive impact of digitalization level on life satisfaction by affecting students' interest.

*H11*: Age plays a moderating role in the positive impact of digitalization level on life satisfaction by influencing students' anxiety.

*H12*: Age plays a moderating role in the positive impact of digitalization level on life satisfaction by affecting students' interest and then affecting their anxiety.

**Figure 1:**
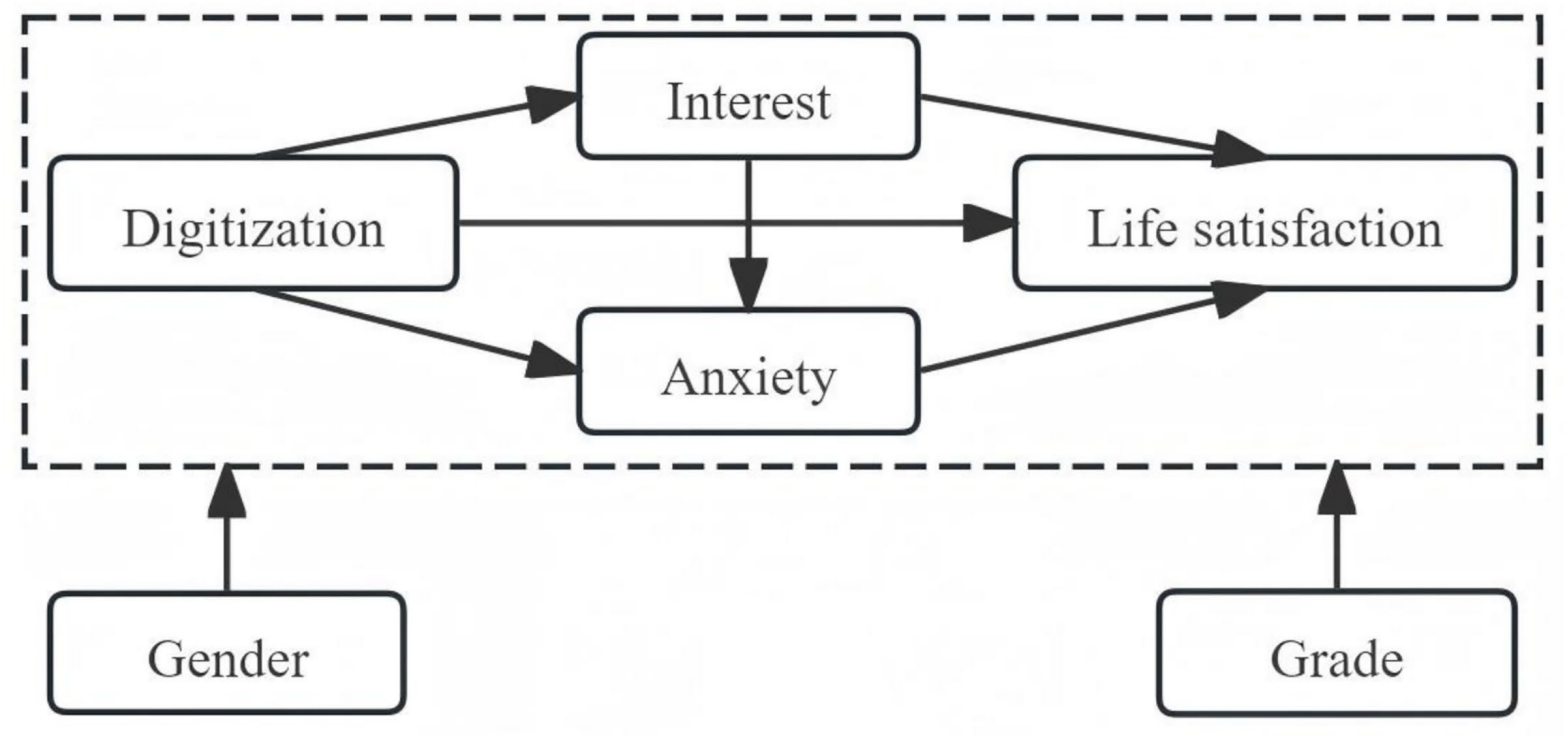
Theoretical model of the impact of digitalization on life satisfaction.

Finally, a theoretical model of the factors affecting life satisfaction from digitalization was constructed, as shown in Figure 1.

## 4 Research design

This study has constructed a theoretical model of the impact of educational digitalization on students’ life satisfaction and will now proceed to further validate and analyze the model using relevant data.

### 4.1 Data sources

The data for this study comes from PISA 2022, an international student assessment program initiated and implemented by the Organization for Economic Cooperation and Development (OECD). Conducted in 2022, this program is the largest and most comprehensive since its inception in 2000. Its core objective is to systematically measure 15-year-old students’ literacy in three core areas: mathematics, science, and reading. It also provides the first standardized global assessment of students’ creative thinking skills. From the perspective of data authority, PISA 2022 offers significant advantages. First, the program is coordinated by the OECD, a prestigious international organization, ensuring the scientific nature and international recognition of the assessment framework. Second, all participating countries and regions use standardized assessment standards, testing tools, and data collection procedures, ensuring data reliability and validity through rigorous quality assurance mechanisms. Finally, PISA results directly influence educational policymaking in participating countries and hold significant policy-leading value. The information and communications technology (ICT) questionnaire, a key component of the PISA assessment system, has evolved over time, reflecting profound shifts in the concept of educational technology assessment. In its early stages (2000–2009), ICT questionnaires primarily focused on the availability of ICT resources, simple frequency surveys of use, and basic ICT skills assessments. During the development phase (2012–2018), PISA data incorporated assessments of ICT usage quality, evaluations of digital learning competence, and analyses of the impact of ICT on learning outcomes. The PISA 2022 ICT questionnaire has undergone comprehensive innovation, marking a significant breakthrough in the transformation of digital assessment. The questionnaire utilizes computer-based assessments of mathematics (CBAM) and adaptive testing technology, enabling real-time data collection and analysis. The assessment content has been significantly expanded to include the frequency and quality of digital device use, experiences with digital learning environments, algorithmic thinking and programming fundamentals, and emerging areas such as digital citizenship. Data collection methods have also been innovative, with process data recording, learning behavior trajectory analysis, and multimodal data fusion providing rich data for in-depth research on students’ digital learning characteristics. By analyzing the data and deleting questionnaires with missing, invalid, and inapplicable data in the selected items, a final data sample of 41,490 was selected for analysis.

### 4.2 Key variables and impacts

This study selected four variables, namely, digitalization level, interest, anxiety, and life satisfaction, for analysis, and selected corresponding questions as observation variables based on the definitions of these variables.

As shown in Table 1, the observed variable for digitalization level is taken from the ICT Questionnaire (IC172), which consists of nine items and is scored on a scale of “1 = Strongly Disagree,” “2 = Disagree,” “3 = Agree,” and “4 = Strongly Agree.” Digitalization level is not a single technical indicator, but rather a systematic indicator encompassing three core dimensions: construction of the hardware and software, digital applications and digital competence of relevant personnel. These nine items precisely cover these three key dimensions, ensuring a comprehensive assessment of the school’s level of digital environment. The digital construction is measured across several dimensions: the extent to which schools provide students with sufficient digital resources, the provision of adequate equipment and internet access to students, whether the network speed is sufficient, the appropriateness of the digital resources provided by schools, and the ease of accessing these resources within student classrooms. The digital application is measured by the extent to which digital resources enhance teaching in schools and the level of technical support provided by the school to assist students in utilizing digital resources. Digital competence is measured by teachers’ ability to use digital devices during the teaching process and their willingness to apply digital devices in instruction.

**Table 1:** Digital questionnaire.

Variable	Serial number	Questionnaire survey questions
Digitalization level	IC172	To what extent do you agree or disagree with the following statements? (Please consider different types of resources, such as desktop computers, laptops, smartphones, and tablet devices, as well as educational software and other digital learning tools.) (Please select one answer per row).
	IC172Q01JA	In school, there are enough digital resources for every student
	IC172Q02JA	My school has enough digital devices and internet access
	IC172Q03JA	The school’s internet speed is fast enough
	IC172Q04JA	Digital resources are functioning properly at my school
	IC172Q05JA	Easy access to digital resources in the classroom
	IC172Q0 6 JA	The digital learning resources provided by our school make learning fun and engaging
	IC172Q07JA	The school provides adequate technical support to help students use digital resources
	IC172Q08JA	Our teachers have the necessary skills to use digital devices in their teaching
	IC172Q0 9 JA	Teachers in our school are willing to use digital resources for teaching

As shown in Table 2, the observed variable for interest is taken from the STQ questionnaire (ST268), which consists of four items and is scored on a scale of “1 = Strongly Disagree,” “2 = Disagree,” “3 = Agree,” and “4 = Strongly Agree.” Subject interest not only reflects students ‘emotional inclination toward a subject but also their confidence in their ability to perform in that subject. These four items effectively cover these two key dimensions.

**Table 2:** Interest questionnaire.

Variable	Serial number	Questionnaire survey questions
Interest	ST268	To what extent do you agree or disagree with the following statements? (Please select one answer per row).
	ST268Q01JA	Mathematics is one of my favorite subjects
	ST268Q03JA	Science is one of my favorite subjects
	ST268Q04JA	Math is easy for me
	ST268Q06JA	Science is easy for me.

As shown in Table 3, the observed variable for learning anxiety was taken from the STQ questionnaire (ST292), which consists of six items and is scored on a scale of “1 = strongly agree,” “2 = agree,” “3 = disagree,” and “4 = strongly disagree.” Learning anxiety is assessed using a reverse scoring method in the PISA questionnaire, that is, the greater the learning anxiety value, the less anxious one is about learning. Learning anxiety is generally understood as a negative emotional state that arises when students face the threat of failure in learning tasks or exams. These items were selected to effectively capture the cognitive, emotional, and physiological manifestations of anxiety, focusing on mathematics, a high-anxiety subject.

**Table 3:** Anxiety questionnaire.

Variant	Serial number	Questionnaire survey questions
Anxiety	ST292	To what extent do you agree or disagree with the following statements? (Please select one answer per row).
	ST292Q01JA	I often worry that math class will be difficult for me
	ST268Q02JA	I get really nervous when I have to do math homework
	ST268Q03JA	I get very nervous when doing math problems.
	ST268Q04JA	I feel helpless when doing math problems.
	ST268Q05JA	I’m worried that I’ll do poorly in math.
	ST268Q06JA	I’m anxious about failing my math test.

As shown in Table 4, the observed variable of life satisfaction is taken from the STQ questionnaire (No. ST016), which has one item and uses a rating scale from “0” to “10.” A score of zero means you are “completely dissatisfied,” while a score of 10 means you are “completely satisfied.”

**Table 4:** Satisfaction questionnaire.

Variant	Serial number	Questionnaire survey questions
Life satisfaction	ST016	The following question asks how satisfied you are with your life on a scale of 0 to 10. A score of 0 means you are not satisfied at all, and 10 means you are completely satisfied. (Please move the slider to the appropriate number).
	ST016Q01NA	Overall, how satisfied are you with your life as a whole these days?

### 4.3 Reliability analysis

This study examined the validity of the measurement model through Confirmatory Factor Analysis (CFA). Table 5 presents the standardized external loading coefficients of each observed variable on the corresponding latent variable. The results show that the “digitalization” latent variable, measured by nine observed indicators (IC172Q01JA to IC172Q09JA), has standardized loading coefficients ranging from 0.724 to 0.820, with an average loading coefficient of 0.772, indicating that these indicators effectively reflect the latent variable of digitalization level. The standardized loading coefficients of the four observed indicators (ST268Q01JA, ST268Q03JA, ST268Q04JA, and ST268Q06JA) for the “interest” latent variable range from 0.608 to 0.880, with an average of 0.735. The loadings of ST268Q03JA and ST268Q06JA are relatively low, but still meet general metrological requirements. The six observed indicators (ST292Q01JA to ST292Q06JA) for the latent variable of anxiety showed standardized factor loadings ranging from 0.687 to 0.840, with an average of 0.754, indicating that each indicator effectively measures the construct of anxiety. The latent variable “life satisfaction” is measured by a single observational indicator (ST016Q01NA), with a standardized loading coefficient of 1.000 (fixed parameter). Although some values are slightly lower, the overall measurement still meets acceptable measurement standards.

**Table 5:** External load.

Variable	Anxiety	Interest	Digitalization	Life satisfaction
IC172Q01JA			0.770	
IC172Q02JA			0.802	
IC172Q03JA			0.726	
IC172Q04JA			0.803	
IC172Q05JA			0.791	
IC172Q06JA			0.756	
IC172Q07JA			0.820	
IC172Q08JA			0.753	
IC172Q09JA			0.724	
ST016Q01NA				1.000
ST268Q01JA		0.853		
ST268Q03JA		0.608		
ST268Q04JA		0.880		
ST268Q06JA		0.624		
ST292Q01JA	0.840			
ST292Q02JA	0.750			
ST292Q03JA	0.781			
ST292Q04JA	0.773			
ST292Q05JA	0.687			
ST292Q06JA	0.702			

To ensure the reliability and validity of the model, we conducted comprehensive testing. Table 6 presents the reliability indices and average variance extracted (AVE) for each latent variable. The Cronbach’s alpha coefficient was used to assess the reliability of the data through SPSS software. By comparing the item results, it provides a more conservative estimate of the internal consistency of the scale (Li, 2022). The Cronbach’s alpha coefficient ranges from 0 to 1. A coefficient below 0.6 indicates poor internal consistency, rendering the questionnaire unacceptable. A coefficient between 0.6 and 0.7 suggests acceptable internal consistency. A coefficient between 0.7 and 0.8 indicates good internal consistency and high questionnaire reliability. A coefficient between 0.8 and 0.9 signifies excellent internal consistency and high questionnaire reliability. The results show that Cronbach’s *α* coefficients for all latent variables were above the acceptable level of 0.7. The Cronbach’s α coefficient for the “digitalization” latent variable was the highest (0.915), indicating that its observed indicators had high internal consistency. The Cronbach’s α coefficients for the “anxiety” latent variable were 0.851, and for the “interest” latent variable were 0.762, both reaching good levels. The results for the comprehensive reliability indices (rho_a and rho_c) were generally consistent with the Cronbach’s α coefficients, further supporting the reliability of the measurement model.

**Table 6:** Reliability and validity test.

Variable	Cronbach’s alpha	Comprehensive reliability (rho_a)	Comprehensive reliability (rho_c)	Average Variance Extracted (AVE)
Anxiety	0.851	0.862	0.889	0.573
Interest	0.762	0.850	0.835	0.565
Digitalization	0.915	0.916	0.930	0.597

The assessment of convergent validity primarily relies on the Average Variance Extracted (AVE) and standardized factor loadings as key indicators. The standardized factor loadings in the questionnaire must exceed the critical value of 0.50 (Anderson and Gerbing, 1988). Higher standardized factor loadings indicate better convergent validity. The Average Variance Extracted (AVE) must be greater than the critical value of 0.50 recommended by Fornell and Larcker (1981). If the AVE is less than 0.50, it indicates that the error variance exceeds the explained variance, meaning the items cannot effectively measure their corresponding latent variable, and the questionnaire is considered unacceptable. A higher AVE signifies better convergent validity (Zhu et al., 2022). In terms of validity, the average variance extracted (AVE) of all latent variables approached the ideal standard of 0.6. The AVE for the “digitalization” latent variable was 0.597, while the AVEs for the “no anxiety” and “interest” latent variables were 0.573 and 0.565, respectively. This indicates that each latent variable explained a significant portion of the variance in its observed indicator. These results demonstrate that the measurement model of this study has good reliability and validity and can be used for subsequent hypothesis testing and model analysis.

### 4.4 Goodness of fit test

As shown in Table 7, the standardized root mean square residual (SRMR) value of this model is 0.060, which is below the critical value of 0.08, indicating that the model residual is small and the difference between the observed data and the model predictions is within an acceptable range. Furthermore, the model’s Normed Fit Index (NFI) is 0.843. According to Bentler and Bonett’s criteria, values closer to 1 indicate better model fit. In summary, the model fit is good.

**Table 7:** Goodness of fit test.

Indicator	Saturation model	Estimation model
SRMR	0.060	0.060
d_ULS	0.753	0.753
d_G	0.254	0.254
Singular variance	60935.325	60935.325
NFI	0.843	0.843

As shown in Table 8, the explanatory power of the latent variables in this study was: R^2^ = 0.213 (adjusted R^2^ = 0.213) for anxiety, 0.017 (adjusted R^2^ = 0.017) for interest, and 0.072 (adjusted R^2^ = 0.072) for life satisfaction, indicating overall weak explanatory power. However, low explanatory power does not necessarily mean that the model loses its analytical value or that the experiment is unfeasible. First, in social science research, particularly in education and psychology, the variance in the dependent variable is often influenced by the interactions of multiple complex factors. Explaining approximately 20% of the variance by a single or a few independent variables is considered to meet the general standard in psychological research (Cohen, 1988). The core objective of this study was to verify the significance of the relationships between variables in the theoretical hypotheses, rather than to maximize explanatory power. Despite the limited explanatory power of some variables, the statistical significance of the key path coefficients still provides empirical support for the research hypotheses.

**Table 8:** Model interpretability.

Variable	R^2^	Adjusted R^2^
Anxiety	0.213	0.213
Interest	0.017	0.017
Life satisfaction	0.072	0.072

## 5 Study results

### 5.1 Model hypothesis testing

This study used the Bootstrap method of SmartPLS and set the sample size to 5,000 to analyze the significance of the path coefficients. This study primarily employs PLS-SEM for data analysis, mainly for the following reasons: As a key modeling approach, PLS-SEM is well-suited for studies with small sample sizes or complex models, as it does not require data to meet the assumption of multivariate normal distribution (Hair et al., 2019). PLS-SEM places greater emphasis on causal-predictive analysis in contexts of lower theoretical development, making it particularly suitable for exploratory models (Pan, 2022) (see Table 9 for details). Based on the advantages of PLS-SEM in areas such as predictive goal alignment, data and model suitability, and practical application convenience, this study employed PLS-SEM for analysis. One of the primary objectives of this study is to explore the predictive relationships among various latent variables (such as digital proficiency, interest, anxiety, and life satisfaction) in digital learning environments and to examine the explanatory power of the theoretical model, rather than conducting rigorous parameter fitting and validation based on pre-existing mature theories. The core strengths of PLS-SEM lie in its strong predictive orientation and its ability to maximize the variance explained in the dependent variables (R^2^values), making it more aligned with the current research objectives compared to Covariance-Based Structural Equation Modeling (CB-SEM). Additionally, PLS-SEM has relatively lenient requirements regarding sample size distribution and data normality, demonstrating greater robustness when handling small to moderate samples and exploratory complex models. This makes it a more suitable analytical tool for the data characteristics of this study. The model in this paper includes four latent variables and twenty observed variables, and PLS-SEM is employed. In short, if the primary purpose of a study is theoretical validation and rigorous goodness-of-fit testing, CB-SEM is typically the preferred choice; however, if the focus is on theory development and predictive application, especially when dealing with novel or complex models, PLS-SEM holds greater advantages. The context of this study aligns more closely with the latter. Finally, this study aims to provide direct and actionable insights for educational practice. The latent variable scores derived from PLS-SEM can be directly utilized for subsequent analyses, which helps to more concretely identify key driving factors and facilitates the proposal of targeted intervention strategies, thereby highly aligning with the applied orientation of this research. Compared to other algorithms, SmartPLS software offers relatively higher estimation accuracy and stability. Additionally, SmartPLS 4 software features a user-friendly interface, straightforward operational procedures, and fast data processing speed. In recent years, due to its distinct advantages, many researchers have utilized it for data processing and analysis. The regression analysis results presented in Table 10 show that all hypotheses were supported by statistical verification. Specifically, digitalization level has a significant positive direct effect on students’ life satisfaction (*β* = 0.032, t = 23.067, *p* < 0.001), which verifies hypothesis H1. Regarding the indirect effect path of digitalization level on life satisfaction through interest, digitalization level has a significant positive impact on life satisfaction through interest (*β* = 0.012, t = 13.005, *p* < 0.001), which is consistent with the expectation of the mediating role of interest in hypothesis H2. At the same time, the level of digitalization can significantly reduce students’ anxiety level (*β* = 0.011, t = 12.459, *p* < 0.001), thereby promoting the improvement of life satisfaction, verifying hypothesis H3. More importantly, the chain mediation path of digitalization level affecting anxiety level through interest and ultimately acting on life satisfaction was verified (*β* = 0.009, t = 17.048, *p* < 0.001), confirming hypothesis H4. This series of empirical results reveals the complex psychological mechanisms by which digitalization affects students’ life satisfaction: digital technology not only directly improves students’ quality of life but also exerts its influence through dual pathways, stimulating positive experiences and alleviating anxiety. The interest-anxiety chain mediation effect provides a novel theoretical perspective for understanding the psychological health-promoting mechanisms of digital education. All validated path effects reached statistical significance (*p* < 0.001), demonstrating that the theoretical model constructed in this study possesses strong statistical power and empirical support, providing important empirical evidence for developing targeted digital education intervention strategies.

**Table 9:** CB-SEM and PLS-SEM.

Item	CB-SEM	PLS-SEM
Research objective	Parameter estimation-oriented	Prediction-oriented
Inference	Optimizes parameter estimation	Maximizes predictive ability
Relationship between latent and observed variables	Only reflective indicators allowed	Both reflective and formative indicators allowed
Data distribution assumption	Requires data to meet normality assumption	No strict requirements; suitable for skewed data
Sample requirement	Large sample size	Small sample size
Model complexity	Simple to moderately complex models	Capable of handling complex models
Theoretical requirement	Requires strong theoretical foundation; supports confirmatory research	Exploratory and explanatory research; no strong theoretical foundation needed

**Table 10:** Regression results and hypothesis testing.

Path	Original sample (O)	Sample mean (M)	Standard deviation (STDEV)	T statistics (|O/STDEV|)	*P*-value	Pass or not
Digitalization → life satisfaction	0.032	0.032	0.001	23.067	0.000	Pass
Digitalization → interest → life satisfaction	0.012	0.012	0.001	13.005	0.000	Pass
Digitalization → anxiety → life satisfaction	0.011	0.011	0.001	12.459	0.000	Pass
Digitalization → interest → Anxiety → life satisfaction	0.009	0.009	0.001	17.048	0.000	Pass

### 5.2 Moderating effect

Guided multi-group analyses were conducted to examine gender and grade differences in the indirect effects of digitalization on life satisfaction (see Table 11). The results indicated no significant gender differences in the indirect effects across all pathways. This finding suggests that the mechanisms through which individuals derive interest, reduce anxiety, and enhance life satisfaction from digital activities are largely consistent regardless of gender.

**Table 11:** Multi-group analysis.

Path	Difference (Female_1 - Male_2)	Two-tailed (Female_1 vs. Male_2) *p*-value	Difference (Middle_1 - Senior_2)	Two-tailed (Middle_1 vs. Senior_2) *p*-value
Digitalization → interest → life satisfaction	0.000	0.986	0.003	0.179
Digitalization → anxiety → life satisfaction	0.000	0.989	0.001	0.692
Digitalization → interest → anxiety → life satisfaction	0.000	0.924	0.004	0.001
Digitalization → life satisfaction	0.000	0.974	0.007	0.014

In contrast, grade differences were significant in some pathways. Here, we divided the study into two groups: junior high school students (grades 7, 8, and 9) and senior high school students (grades 10, 11, and 12). Specifically, in the mediation path of “digitalization → interest → anxiety → life satisfaction,” the indirect effect for the junior high school group (Middle_1) was significantly greater than that for the senior high school group (Senior_2), with a difference of 0.004 and a two-tailed *p*-value of 0.001. This suggests that junior high school students are more likely to improve their life satisfaction through interest and emotional stability. Furthermore, in the direct effect path of “digitalization → life satisfaction,” the effect for the junior high school group was significantly stronger than that for the senior high school group, with a difference of 0.007 and *p* = 0.014. However, while positive differences were present in other pathways, they did not reach statistical significance.

### 5.3 Discussion

#### 5.3.1 Digitalization level has a positive impact on students’ life satisfaction

The regression analysis results showed that digitalization level had a significant positive direct effect on student life satisfaction (*β* = 0.032, t = 23.067, *p* < 0.001), confirming hypothesis H1. This finding is highly consistent with the core concept of the Technology Acceptance Model. Davis (1989) pointed out that when individuals perceive technology as easy to use and useful, they will have more positive attitudes and experiences using it. Students with higher levels of digital literacy are better able to fully utilize digital resources and tools, thereby enhancing their personal learning outcomes and life satisfaction. From the perspective of the Technology Acceptance Model (TAM), an improvement in digital literacy significantly increases students’ perceived ease of use of technological tools. When individuals can skillfully operate AI-assisted systems, online learning platforms, or digital collaboration tools, the technology itself no longer poses a cognitive barrier but instead becomes a seamlessly integrated cognitive extension of their daily learning processes. This smooth technological interaction reduces psychological friction during task execution, shifting focus from “how to use the tools” to “how to accomplish tasks,” thereby improving learning efficiency and optimizing emotional states. For example, students with advanced search skills can precisely locate academic literature using Boolean operators, avoiding inefficient navigation through vast amounts of information. Such efficient operations directly enhance their positive evaluation of technological systems. Meanwhile, perceived usefulness, as another core construct, reflects students’ subjective judgment of whether technology can tangibly enhance their performance (Arpaci and Basol, 2020). High levels of digital literacy enable learners to access high-quality educational resources beyond temporal and spatial constraints, such as participating in MOOC courses, accessing open databases, or utilizing intelligent recommendation systems to discover personalized learning content.

Digital education offers students flexible learning options, enabling them to learn at their own pace and according to their needs. This flexibility has a positive impact on students’ mental health. Digital education can combine positive psychology with high-tech educational methods to create a more personalized learning experience for students (Graesser et al., 2022). Through information technology-enabled courses, students can access learning resources anytime and anywhere, reducing the pressure caused by time and space constraints. Students can also explore and learn more independently, achieving innovative thinking (Hasni and Sampelolo, 2024; Fadhilah et al., 2021; Suwastini et al., 2023). For instance, students can learn through online courses at any time and place, progressing at their own pace (Fang, 2015), without being constrained by time, location, or other conditions, which significantly reduces their academic pressure and burden. Moreover, as for generative artificial intelligence, students can also access it at any time, often with free access, greatly enhancing the convenience and effectiveness of its use for students (Leng, 2024). When students are able to independently choose their learning content, pace, and methods, their intrinsic motivation is stimulated, their need for autonomy is satisfied, and this in turn promotes their mental health.

Digital education also effectively enhances students’ sense of achievement by providing immediate feedback and recognition of accomplishments. From the perspective of information processing theory, digital literacy essentially reshapes the structure of students’ ability to process environmental stimuli. In modern educational contexts, information overload has become a common challenge. Students with strong digital literacy possess more effective strategies for filtering, organizing, and integrating information, enabling them to maintain a higher level of cognitive control in complex environments (Yan et al., 2016). They can use reference management software (such as Zotero or EndNote) to build personal knowledge databases, achieving long-term storage and rapid retrieval of information through tag-based categorization. They can also utilize mind-mapping tools to visualize fragmented knowledge, fostering deeper comprehension and meaning construction. Such efficient resource management capabilities effectively alleviate anxiety triggered by information uncertainty and enhance an individual’s adaptability and sense of control in the knowledge society. More importantly, each successful problem-solving experience serves as a significant source of self-efficacy (Hatlevik et al., 2018). Bandura points out that mastery experiences constitute the most powerful pathway for shaping efficacy beliefs (Jiang et al., 2025). When students can visualize their progress and achievements through digital tools, their sense of competence is enhanced, which positively impacts their mental health (Abdous, 2019; Lister et al., 2024; Shareefa et al., 2025). Through online communities and collaborative learning platforms, students can build positive social connections, satisfy their need for belonging, and gain a sense of competence by completing learning tasks, thereby promoting overall mental health. Digital tools also provide students with a wealth of mental health resources and support. Websites and mobile apps can provide information on well-functioning, learning, and community participation, which can help students cope with psychological challenges.

It’s important to note that the positive impact of digital education on mental health requires moderation to maximize its benefits. Research shows that moderate digital media use has limited impact on psychological well-being, while excessive use can have negative consequences (Twenge, 2019). Therefore, educators should design balanced digital education programs to ensure that the use of technology tools serves students’ mental health needs rather than becoming a new source of stress. Through carefully designed digital education strategies, we can fully leverage the benefits of technology and create a healthier and more positive learning environment for students. Meanwhile, educational institutions should regard the cultivation of digital literacy as a prerequisite component of mental health interventions. Through systematic skills training, students’ technological self-efficacy and information management efficiency can be enhanced. This approach helps alleviate technology-related anxiety and cognitive load at the source, thereby creating a learning and living environment where students experience a greater sense of control and accomplishment.

#### 5.3.2 Digitalization level has a positive impact on life satisfaction by affecting students’ interest

The indirect effect of digitalization on life satisfaction through interest was significantly confirmed (*β* = 0.012, t = 13.005, *p* < 0.001), supporting Hypothesis H2. Digital tools, through their interactivity, personalization, and multimedia features, effectively stimulate students’ learning interest and exploration motivation. With abundant online resources and tools, students can utilize them for personalized learning and lifelong learning, significantly enhancing their learning motivation and interest. Through online interaction, students can receive timely answers to their questions (Li et al., 2021), which substantially boosts their learning enthusiasm and engagement. According to self-determination theory, interest, as a key component of intrinsic motivation, is a key factor influencing individual well-being (Deci and Ryan, 1985). The rich and diverse learning resources and immediate feedback mechanisms provided by digital learning environments can meet the needs of students with different learning styles, enhancing their sense of engagement and achievement. Multiple intelligence theory further supports this finding. Gardner emphasizes that different students have different intelligence strengths, and digital technology provides a platform for learners of various intelligence types, such as linguistic intelligence, logical-mathematical intelligence, and even visual–spatial intelligence, to demonstrate and develop their talents (Gardner and Kraut, 1984). For instance, learners can adjust their progress according to their own pace, independently select content difficulty, or integrate cross-disciplinary learning resources. This heightened sense of control directly addresses the core proposition emphasized by Self-Determination Theory (SDT), that “behavior should stem from intrinsic volition rather than external compulsion” (Ryan and Deci, 2017). When individuals perceive their learning decisions as driven by their own will, tasks transform from external demands into internal pursuits, thereby spontaneously generating sustained and stable learning interest (Güleç, 2024). When students’ interest is effectively aroused, this positive motivation encourages them to shift their attention from external distractions to the learning task itself, making it easier for them to enter the “flow” state proposed by Csikszentmihalyi. In a state of “flow,” students experience a perfect match between challenge and skill, achieving a high degree of immersion and intrinsic enjoyment, significantly enhancing the efficiency of their cognitive engagement. This positive emotional experience, fostered by interest and flow, extends beyond learning to generate positive spillover effects through the expansion and construction of emotions (Guo, 2020; Zhou et al., 2022). Positive emotions can broaden an individual’s cognitive and behavioral scope, making students more inclined to adopt proactive coping strategies when facing other challenges in life. Ultimately, through the accumulation and transfer of these emotions, overall life satisfaction can be significantly improved. For example, traditional paper-and-pencil assessments often have delayed feedback cycles, which diminishes the motivating effect of performance information. In contrast, intelligent learning systems can provide real-time evaluation of responses, dynamic competency mapping, and badge reward systems, allowing students to clearly perceive their own progress trajectory. This timely, objective confirmation of ability effectively strengthens self-efficacy beliefs and satisfies fundamental psychological needs for competence growth (Cronin et al., 2020). Competency experiences in digital environments are not limited to knowledge acquisition alone; they also encompass the development of new literacies such as technical operational proficiency and information integration skills. The accumulation of these multidimensional competencies further consolidates an individual’s sense of comprehensive competence, serving as a key pillar for sustaining long-term learning interest (Lee and Chen, 2017). Mechanisms such as online discussion forums, virtual group projects, and peer assessment systems break down spatial and temporal barriers, promoting frequent interactions between teachers and students and among students themselves. By engaging in collaborative knowledge building, students gain a sense of belonging and recognition, forming a learning community with shared goals (Leversen et al., 2012). Especially in remote or blended learning models, digital platforms become vital links for maintaining social connections, preventing the psychological alienation that can arise from isolated learning. When students feel understood and supported through social connections within digital spaces, their learning experience gains emotional warmth and a greater sense of meaning, thereby significantly enhancing their subjective well-being. For education, this highlights the central importance of motivational design in digital education. This finding provides a new perspective for educational research: when evaluating the value of technology integration, the effectiveness of fostering intrinsic motivation should be considered a key indicator of psychological well-being. This also requires educators, when deploying digital tools, to look beyond their function as mere information carriers and instead view them as enablers for satisfying fundamental psychological needs.

#### 5.3.3 Digitalization level has a positive impact on life satisfaction by affecting students’ anxiety

The results show that digital literacy positively impacts life satisfaction by reducing anxiety (*β* = 0.011, t = 12.459, *p* < 0.001), confirming Hypothesis H3. This finding robustly supports the core proposition of the Stress and Coping Theory, which posits that an individual’s level of coping with environmental challenges significantly influences their perceived stress and psychological well-being (Lazarus and Folkman, 1984). In the digital age, students face the potential challenges of rapid technological advancement, information overload (Chen et al., 2025), and the “digital divide.” A lack of corresponding digital skills often leads to increased technological anxiety and learning pressure, manifested as resistance to using new tools, frustration due to operational errors, and cognitive overload resulting from the inability to effectively filter information in digital environments (James et al., 2021; Masrek and Baharuddin, 2023). Students with higher digital proficiency possess stronger technological adaptability and problem-solving skills, enabling them to navigate digital learning tasks with ease. Students with higher digital proficiency possess stronger technological adaptability and problem-solving abilities, enabling them to approach digital learning tasks with ease. They are capable of transforming technical operations from “conscious efforts” into “automatic processes,” which allows them to handle digital learning tasks more smoothly (Harnani et al., 2021), effectively reducing the cognitive load caused by technical barriers (Özeren, 2023). This increased efficiency and sense of control fundamentally reduces the anxiety associated with a sense of loss of control. The broaden-and-build theory of emotion further explains this mechanism. Negative emotions narrow an individual’s scope of thinking and behavior, whereas positive emotions enhance their cognitive and behavioral capacity (Fredrickson, 2001). Therefore, by reducing the negative emotional state of anxiety, digital proficiency effectively removes this constraint, creating a more positive psychological environment for students and enhancing their resilience. This psychological resilience is not only reflected in learning as a stronger ability to resist frustration, but also spills over to a wider range of life areas, enabling individuals to deal with daily challenges more optimistically and positively, ultimately promoting an improvement in life satisfaction.

#### 5.3.4 Digitalization level affects students’ anxiety by affecting their interest, thus having a positive impact on life satisfaction

In our research, the chain mediation path, through which digitalization level influences anxiety levels via interest and ultimately life satisfaction, was significantly confirmed (*β* = 0.009, t = 17.048, *p* < 0.001), confirming Hypothesis H4. This chain mediation effect reveals the complex psychological mechanism by which digitalization level influences students’ life satisfaction, demonstrating the dynamic regulatory relationship between positive and negative emotions. According to emotion regulation theory, positive emotions can effectively regulate and mitigate the impact of negative emotions (Gross, 1998). When students develop a strong interest in digital technology, they enter a positive emotional state. This state not only enhances attention and cognitive engagement, but also makes it easier for them to experience a state of “flow” (Csikszentmihalyi, 1991). In this flow experience, an individual’s entire mental resources are focused on the task itself, resulting in a temporary loss of awareness of time and self (Parvizi-Wayne et al., 2024). This high level of immersion effectively blocks excessive focus and rumination on learning stressors and potential anxiety at a cognitive level (Pace, 2007). Simultaneously, this high level of immersion can promote the secretion of neurotransmitters such as dopamine in the brain. The dopamine system plays a central role in reward, motivation, and stress regulation. Therefore, the release of this neurotransmitter not only reinforces the learning behavior itself but also reduces the perception of stress and anxiety triggered by learning tasks on a physiological level by raising the individual’s threshold. This finding offers a new theoretical lens for understanding the mechanisms through which digital education promotes psychological well-being. This indicates that digital technology affects student well-being not merely through direct pathways, but more importantly by triggering a mediated chain of psychological processes: fostering positive emotions → buffering negative emotions → enhancing life satisfaction. In other words, interest is not only a state of learning but also functions as a form of psychological capital. It helps students build positive cognitive schemas and emotional reserves within digital environments, thereby bolstering their psychological resilience.

#### 5.3.5 Regulatory effect of gender and grade

The present study found that the moderating effect of gender on the relevant paths was not significant. This aligns with many current studies. Numerous existing studies indicate that as society develops, the differentiation in social roles and status between men and women is gradually diminishing. The differences between men and women in terms of usage patterns, intensity, and other aspects of digital technology application are also narrowing (Fang and Cai, 2025). Consequently, the impact of digital literacy on life satisfaction is no longer significant. Traditional perspectives generally held that men exhibit higher self-efficacy in digital technology use, while women are more susceptible to information overload and computer anxiety. However, this study failed to validate the role of gender as a moderating variable, which may reflect a fundamental shift occurring in contemporary digital environments. With the advancement of educational equity policies, the widespread adoption of information technology courses, and the gradual erosion of gender stereotypes in society, the gap between male and female students in accessing and mastering digital tools may have significantly narrowed. Particularly among “digital native” groups, regardless of gender, individuals have been immersed in smart devices and online environments since childhood. This universal exposure to technology may have weakened the expression of traditional gender differences (Bellini et al., 2016). Secondly, although some earlier studies did report an advantage for men in computer self-efficacy, an increasing number of cross-national surveys in recent years have revealed a more complex picture. For example, data from ICILS 2013 showed that in most participating countries, 14-year-old girls outperformed boys in computer and information literacy assessments (Punter et al., 2016). This suggests that gender differences are neither unidirectional nor universal; their direction and magnitude are highly dependent on specific skill dimensions, cultural contexts, and developmental stages. The findings of this study echo this trend, reminding us to move beyond simplistic binary frameworks like “male superiority/female inferiority” and instead focus on how task types, usage motivations, and situational factors collectively shape digital behavioral patterns. Although the hypothesis was not confirmed, this result challenges the habitual thinking that treats gender as a default moderating variable and emphasizes the need to reassess its explanatory power within the modern digital ecosystem. It supports a more nuanced perspective: individual differences (such as personality traits, prior experience, learning styles) may be more effective predictors of digital adaptation than biological sex (Luo et al., 2012). This also aligns with the developmental direction of social cognitive theory, which posits that the formation of self-efficacy is the result of multiple interactions rather than being determined by a single variable.

The mechanism underlying these age differences lies in fundamental differences in cognition, emotions, and environmental pressures faced by students at different stages of development. This provides new evidence for developmental psychology theory. The results confirm Erikson’s theory of psychosocial development, which states that individuals at different developmental stages face different core tasks and psychological needs (Niu and Guo, 2021). Students in middle school are at a stage of identity exploration, during which they have an urgent need to explore their self-image, interests, preferences, and sense of social belonging. Digital platforms provide a low-risk, high-freedom space for identity experimentation—expressing individuality through social media, building connections by participating in virtual communities, and trying out different roles in games. These behaviors all help alleviate identity anxiety, strengthen self-identity, and thereby enhance life satisfaction. Junior high school students are in a period of identity exploration and are more sensitive and receptive to novelty. Middle school students are in a period of identity exploration, and this age group exhibits heightened sensitivity and receptiveness to novel experiences. This enables multimedia-rich and highly interactive digital teaching methods to efficiently stimulate their curiosity and learning interest (Aggarwal et al., 2024; Grüning and Krueger, 2024). This stimulated interest does not merely remain at the level of superficial enjoyment but can be more effectively translated into positive emotional feedback, forming a positive and virtuous cycle during the learning process, thereby significantly enhancing their overall life satisfaction. In contrast, senior students enter a period of academic achievement-oriented conflict between “intimacy and loneliness” or “diligence and inferiority,” and their learning anxiety has reached a “ceiling effect.” In this entrenched pressure environment, the small increase in interest brought about by digitalization becomes insignificant against the backdrop of intense environmental pressure, and its positive emotional spillover effect is diluted by the competitive environment and the focus on grades (Khrapov and Baeva, 2022; Shang et al., 2023). Therefore, the same digital intervention’s effect on improving life satisfaction through interest and emotional pathways is suppressed in high school students, resulting in insignificant differences in the mediation effect.

## 6 Limitation

While this study has conducted in-depth theoretical and empirical analyses of the impact of educational digitalization on students’ life satisfaction, it also has several potential limitations due to factors such as data constraints, which point to avenues for future academic exploration. First, the data is limited by its cross-sectional design. While using cross-sectional PISA data collected at a single time point effectively validates correlations and mediating mechanisms between variables, it is difficult to directly rule out the possibility of reverse causality (for example, students with high life satisfaction may have more positive attitudes toward digital education environments and thus demonstrate higher levels of digital proficiency). More importantly, cross-sectional data limit our ability to track the long-term changes and dynamic adaptation processes in the impact of digital interventions or environmental changes on student well-being. Future research should prioritize longitudinal follow-up designs (such as cross-lagged models) to more rigorously examine the causal mechanisms between digitalization and student well-being and assess the persistence of these effects. Second, the measurement methods and indicator construction of the variables present certain limitations. The core variables of this study, including life satisfaction, interest, and anxiety, all rely on self-reported responses from students, which may lead to social desirability effects or recall bias. Furthermore, the presence of omitted variables in the model, such as teacher support for digital teaching, family environmental factors, or the school’s innovative climate, may also bias the model estimates. Future research should consider these multi-level factors. Finally, in terms of model explanatory power, while the core path in this study is significant, the overall R^2^ value is low, indicating that the model captures only a limited portion of the variance in the variables. This underscores the multifaceted complexity of student life satisfaction, and unmeasured variables may exert stronger effects. This reminds us that the impact of digitalization is not an isolated mechanism, but rather part of a broader ecosystem.

## 7 Conclusion and implication

This study constructed a theoretical model of the impact of educational digitalization on students’ life satisfaction and conducted an in-depth empirical analysis of this model using PISA 2022 data. The findings derived on the influence of educational digitalization offer informative perspectives and a foundation for guiding its future development and enhancing student life satisfaction.

### 7.1 Theoretical influence

At present, although there have been scattered theoretical analyses on the impact of educational digitization on life satisfaction, there is still a lack of empirical research that systematically examines the influence of “educational digitization” as a core independent variable on the core dependent variable “life satisfaction.” This study constructs a theoretical model to explore the impact of educational digitization on life satisfaction and conducts an in-depth empirical investigation based on international PISA data, addressing the existing gap in empirical research. Furthermore, existing studies have yet to sufficiently deepen the analysis of the mechanisms through which educational digitization affects life satisfaction, including the roles of mediating and moderating variables. This study introduces students’ learning interest and anxiety as mediating variables, while incorporating gender and grade level as moderating variables, to thoroughly analyze the mechanisms by which educational digitization influences students’ life satisfaction. The research content is unique and enhances the understanding of how educational digitization affects students’ life satisfaction, providing theoretical foundations and data support for the further advancement of educational digitization and the improvement of student satisfaction. The study confirmed a significant positive direct effect of digitalization on life satisfaction, strongly supporting the applicability of the Technology Acceptance Model in education. By validating the significance of interest, anxiety, and the “interest-anxiety” chain mediation pathway, the study significantly enriches the application scenarios of self-determination theory and emotion regulation theory. Digitalization is not only a vehicle for knowledge transfer but also a crucial environmental resource for stimulating intrinsic motivation and alleviating psychological stress. The existence of the “digitalization → interest → anxiety → life satisfaction” chain mediation effect is particularly noteworthy, revealing a dynamic psychological transformation process: positive emotions not only independently enhance well-being but also serve as a powerful “buffer,” effectively offsetting the negative impact of negative emotions on life satisfaction. Furthermore, the study did not find significant gender differences, but did reveal a significant moderating effect of grade level, with junior high students being more likely to be curious about new things and to experience greater pleasure in digital environments than high school students.

### 7.2 Practical implications

Although the measured effects are modest, they suggest that focused efforts to enhance students’ interest and reduce their learning anxiety in the context of digital applications can contribute meaningfully to student well-being. The aforementioned research offers significant practical implications. First, teachers should place greater emphasis on incorporating designs and implementations that stimulate and sustain students’ learning interest in digital education applications. For teachers, the research findings further underscore the central importance of stimulating and sustaining students’ learning interest in instructional design. In digital instructional practices, teachers should not simply view technology as a tool for information delivery or task assignment, but should integrate it into the design of exploratory, creative, and social learning activities. Instructional design should fully consider students’ cognitive developmental stages and psychological needs, and engage students in active learning within real or simulated contexts through contextualization, gamification, and project-based approaches. For example, using virtual reality (VR) technology to create immersive experimental environments in science instruction, allowing students to conduct chemical reactions or astronomical observations in a virtual space, can enhance the learning experience and boost learning motivation. Similarly, introducing programming projects into information technology courses, allowing students to design mini-games, animations, or applications, can not only strengthen their computational thinking skills but also make the learning process more fulfilling and empowering. Through this virtuous cycle of “interest-driven learning, independent exploration, and positive feedback,” teachers can promote students’ academic development while indirectly improving their mental health and learning well-being. Second, school xxistrators should further foster an inclusive, low-anxiety digital learning ecosystem. For school xxistrators, the research highlights the importance of constructing an inclusive, low-anxiety digital learning environment. Schools should ensure the fairness and sustainability of education’s digital transformation through institutional means, ensuring that all students have equal access to high-quality digital devices, online resources, and learning platforms. Furthermore, schools should establish a comprehensive technical support system and provide ongoing digital literacy training to reduce the “digital anxiety” caused by technological barriers. At the management level, schools should rationally manage students’ screen time to prevent visual fatigue, information overload, and social isolation caused by excessive reliance on online learning. Furthermore, schools should encourage the development of “online + offline” hybrid learning models, leveraging offline interactions to strengthen interpersonal communication and emotional connections, thereby striking a balance between technological convenience and psychological well-being. Only when schools transform the concept of “integrating technology into education” into “building a people-centered digital ecosystem” can the healthy development of digital education truly be achieved. Finally, when evaluating the effectiveness of digital projects, education authorities should incorporate emotional indicators such as learning interest and technology anxiety levels into the assessment criteria, rather than focusing solely on test scores and usage duration. Integrating emotional indicators into the evaluation of digital education projects signifies a profound shift in the assessment paradigm—from an “efficiency-oriented” approach to one that is “student development-oriented.” Traditional metrics centered on scores and usage time can only reflect the breadth of technology adoption and the efficiency of knowledge delivery, yet they fail to capture the deeper impact of technology on students’ learning experiences and internal states. Emotional indicators like learning interest and technology anxiety serve as critical bridges connecting external stimuli with internal cognition: heightened interest acts as the driving force for deep engagement and sustained learning, whereas widespread technology anxiety can directly undermine cognitive resources, turning digital tools into new sources of stress. Therefore, the evaluation system must integrate multimodal data—such as survey questionnaires, behavioral analysis, and affective computing—to construct a comprehensive framework that measures both “cognitive gains” and “emotional experiences.” This shift not only helps identify and mitigate the risks of “technology-induced stress” but also guides project design back to the essence of education—prioritizing students’ mental health, learning quality, and holistic development in the digital age. Ultimately, it propels digital education from merely pursuing “accessibility” toward achieving high-quality development that ensures “effective use and meaningful growth.” Third, focus on differentiated digital application strategies. In the application of educational digitalization, emphasis should be placed on differentiated approaches. For instance, for junior high school students, more engaging and gamified design applications can be adopted, while for senior high school students, personalized recommendation systems can be utilized to alleviate their academic pressure. Junior high school students are in a critical period of transitioning from concrete to formal operations. They are highly curious but have relatively limited sustained attention, with a strong need for social recognition and immediate feedback. Therefore, mere digital presentation is far from sufficient, and more educational fun design should be incorporated. For example, narrative-based and exploratory learning scenarios can be constructed, such as designing history courses as time-travel adventures or integrating the learning of physics laws into solving virtual-world physics puzzles. For senior high school students, “personalized recommendation systems” can be employed to provide precise empowerment and support independent deep learning. Senior high school students’ abstract thinking tends to mature, and they face pressure with clear learning goals. Individual differences—such as knowledge foundations, thinking preferences, and learning paces—become significantly pronounced. Their core needs are efficiency and precision. This approach is not only intended to “alleviate academic pressure” but also to cultivate students’ metacognitive abilities and self-directed learning skills, enabling them to become mature learners capable of self-planning and self-monitoring. For digital education product developers, the research indicates that students at different educational stages exhibit significant differences in learning motivation and psychological characteristics. Therefore, product design should adhere to the principle of developmental appropriateness. For junior high students, developers should focus on product fun, exploratory, and gamified experiences to fully tap into their curiosity and desire for new experiences. Interactivity can be enhanced through task rewards, plotted storylines, or the company of virtual characters, allowing students to experience continuous learning pleasure in the process of “interest-involvement-achievement,” thereby promoting a positive linkage between learning interest and life satisfaction. For high school or college students, product design should focus more on practicality, efficiency, and goal orientation. For example, it can provide accurate academic diagnosis systems based on learning analysis, personalized review path recommendations, and career planning assistance tools to help students better regulate their emotions, plan their time, and improve their self-efficacy amidst the pressure of exams and future uncertainty. Developers should also pay attention to the design of user psychological feedback mechanisms, establish a two-way monitoring model for learning achievement and emotional health, avoid increased student anxiety due to excessive competition or complex functions, and maximize the positive effects of the “interest-anxiety-satisfaction” chain. Fourth, strengthen teachers’ emotional regulation and psychological support functions through professional training. To address the challenges in the emotional dimension of the digitalization in education, it is essential to fundamentally reinforce and reshape teachers’ roles in emotional regulation and psychological support through systematic and ongoing professional training, enabling them to become the “emotional anchors” and “development coaches” within students’ digital learning ecosystems. This deepening is not merely about adding a skill but requires teachers to transition from “knowledge transmitters” to “collaborators in holistic development.” Specifically, training should equip teachers with keen “digital emotional insight,” enabling them to accurately identify subtle emotional signals—such as technological anxiety, information fatigue, or even digital alienation—displayed by students when confronted with complex interfaces, information overload, or frustrations in human-computer interactions. They should also master preliminary psychological support techniques, such as cognitive reappraisal and active listening, to provide immediate intervention. More critically, teachers must be empowered to become “proactive architects” of digital learning environments. They should actively utilize online collaboration platforms to design project-based, community-oriented learning tasks and intentionally foster highly trusted, strongly connected online learning communities. Within such communities, peer support, shared achievements, and social validation can effectively meet students’ needs for belonging and relatedness, thereby transforming the pressure of atomized individual learning into collectively supported wisdom. Simultaneously, teachers must also act as “digital wellness mentors,” moving beyond the technical application of tools to guide students in scientifically utilizing digital resources—such as mindfulness apps, emotion journaling tools, or immersive relaxation content—for self-regulation and emotional management, thereby cultivating the psychological resilience crucial in the digital age. Ultimately, this series of functional enhancements aims to construct a new educational scenario where “technological empowerment” and “humanistic care” are deeply integrated, ensuring that the digitalization process not only enhances cognitive efficiency but also nurtures students’ mental health and sustainable development capabilities.

To deepen research on educational digitalization and students’ life satisfaction, future studies can be further expanded in the following directions: First, refine the impact mechanism model. Building on existing research, variables such as teachers’ digital teaching support, family digital environment, and school innovation climate should be incorporated to develop multilevel mediation or moderation models. This will more systematically reveal the pathways and boundary conditions through which educational digitalization influences students’ life satisfaction, with particular attention to its complex role in the process of “technology application–psychological experience–life perception.” Second, promote data and methodological integration. By combining subjective reports with objective data (e.g., digital platform usage logs, physiological indicators, or behavioral observations), mixed-methods research designs can be employed to capture the actual usage patterns of educational digitalization and its emotional and social impacts across multiple dimensions. This approach will enhance the validity and explanatory power of the research. Third, conduct longitudinal tracking and process studies. Design cross-temporal tracking surveys to examine the dynamic effects of educational digitalization on students’ life satisfaction, distinguishing between short-term adaptation and long-term outcomes. Special attention should be paid to the interaction between critical developmental stages and the timing of technological interventions, providing a basis for timely educational interventions. Fourth, emphasize heterogeneity across developmental stages. Tailored research frameworks and measurement tools should be designed for students at different educational levels, such as primary, junior high, and senior high school, considering their cognitive and emotional development characteristics. This will help explore age-related differences and mechanistic variations in the impact of educational digitalization, providing empirical support for building a digital education ecosystem that meets developmental needs. Through these advancements, research will not only provide a more comprehensive and profound understanding of the relationship between educational digitalization and adolescent well-being but also offer a more thorough scientific reference for promoting human-centered digital education practices and mental health support policies.

## Data Availability

The datasets presented in this study can be found in online repositories. The names of the repository/repositories and accession number(s) can be found at: https://www.oecd.org/pisa/data/2022database/.
